# Expression of truncated Kir6.2 promotes insertion of functionally inverted ATP-sensitive K^+^ channels

**DOI:** 10.1038/s41598-021-00988-y

**Published:** 2021-11-02

**Authors:** Benjamin A. Heitz, Robert Bränström, Wei Yang, Yiding Huang, Tilo Moede, Ingo B. Leibiger, Barbara Leibiger, Liu Qi Chen, Jia Yu, Shao-Nian Yang, Olof Larsson, S. Scott Saavedra, Per-Olof Berggren, Craig A. Aspinwall

**Affiliations:** 1 Department of Chemistry and Biochemistry, University of Arizona, Tucson, AZ 85721 USA; 2 BIO5 Institute and Department of Biomedical Engineering, University of Arizona, Tucson, AZ 85721 USA; 3 The Rolf Luft Research Center for Diabetes and Endocrinology, Karolinska Institutet, Karolinska University Hospital, 171 76 Stockholm, Sweden; 4 Endocrine and Sarcoma Surgery Unit, Department of Molecular Medicine and Surgery, Karolinska Institutet, Karolinska University Hospital, 171 76 Stockholm, Sweden

**Keywords:** Protein transport, Membrane trafficking

## Abstract

ATP-sensitive K^+^ (K_ATP_) channels couple cellular metabolism to electrical activity in many cell types. Wild-type K_ATP_ channels are comprised of four pore forming (Kir6.x) and four regulatory (sulfonylurea receptor, SURx) subunits that each contain RKR endoplasmic reticulum retention sequences that serve to properly translocate the channel to the plasma membrane. Truncated Kir6.x variants lacking RKR sequences facilitate plasma membrane expression of functional Kir6.x in the absence of SURx; however, the effects of channel truncation on plasma membrane orientation have not been explored. To investigate the role of truncation on plasma membrane orientation of ATP sensitive K^+^ channels, three truncated variants of Kir6.2 were used (Kir6.2ΔC26, 6xHis-Kir6.2ΔC26, and 6xHis-EGFP-Kir6.2ΔC26). Oocyte expression of Kir6.2ΔC26 shows the presence of a population of inverted inserted channels in the plasma membrane, which is not present when co-expressed with SUR1. Immunocytochemical staining of intact and permeabilized HEK293 cells revealed that the N-terminus of 6xHis-Kir6.2ΔC26 was accessible on both sides of the plasma membrane at roughly equivalent ratios, whereas the N-terminus of 6xHis-EGFP-Kir6.2Δ26 was only accessible on the intracellular face. In HEK293 cells, whole-cell electrophysiological recordings showed a ca. 50% reduction in K^+^ current upon addition of ATP to the extracellular solution for 6xHis-Kir6.2ΔC26, though sensitivity to extracellular ATP was not observed in 6xHis-EGFP-Kir6.2ΔC26. Importantly, the population of channels that is inverted exhibited similar function to properly inserted channels within the plasma membrane. Taken together, these data suggest that in the absence of SURx, inverted channels can be formed from truncated Kir6.x subunits that are functionally active which may provide a new model for testing pharmacological modulators of Kir6.x, but also indicates the need for added caution when using truncated Kir6.2 mutants.

## Introduction

Adenosine triphosphate (ATP)-sensitive K^+^ (K_ATP_) channels are present in a wide range of tissues^1–4^ including pancreatic islet cells^4–8^, heart^9–11^, skeletal muscle^12^, vascular smooth muscle^11,13^, and brain^14^. K_ATP_ channels serve to couple the metabolic state of the cell to electrical activity^6^. As such, K_ATP_ channels play key roles in regulating diverse biological functions such as insulin secretion^6,15^, cardiac action potentials^16,17^, ischemic preconditioning^18,19^, and blood pressure^20^.

K_ATP_ channel structure has been investigated in detail in a range of tissues^1,3,4,21^. Cloning and reconstitution of functional K_ATP_ channels and channel variants have illuminated key advances in K_ATP_ channel structure–function relationships^7^. Structurally, K_ATP_ channels are heterooctamers comprised of four pore forming (Kir6.x) subunits from the family of small inwardly rectified K^+^ channels and four regulatory subunits (SURx) from the sulfonylurea receptor family^1,3,4,21,22^. While most physiological and pharmaceutical ligand sensitivity is imparted by the SURx subunits^1,3,4,23^, ATP, phosphoinositides and long-chain coenzyme A (LC-CoA) esters act via the Kir6.x subunit^3,24–29^. Among the most studied K_ATP_ channels are those expressed in the insulin secreting pancreatic β-cell, where the K_ATP_ channel is comprised of Kir6.2 and SUR1^1,29–31^. A range of diseases are associated with either mutation and/or improper trafficking of SURx or Kir6.2 subunits, including type 2 diabetes mellitus^8,32,33^, and persistent hyperinsulinemic hypoglycemia of infancy (PHHI)^3,20,34–36^.

Functional expression of wild-type Kir6.2 in the plasma membrane of model organisms requires co-expression with SURx^7,29^. Detailed investigation of this phenomenon revealed the presence of an endoplasmic reticulum (ER) retention signal comprised of a three amino acid (RKR) sequence on both Kir6.2 and SURx^37^. Proper trafficking of the K_ATP_ channel requires that all RKR sequences, from both subunits, be shielded, which occurs during assembly of the channel complex within the ER^37^. Further, only functional K_ATP_ channels in the proper stoichiometric ratio in the ER can sufficiently mask the RKR retention signal and facilitate export to the Golgi apparatus and eventually the plasma membrane^37^.

**Figure 1:**
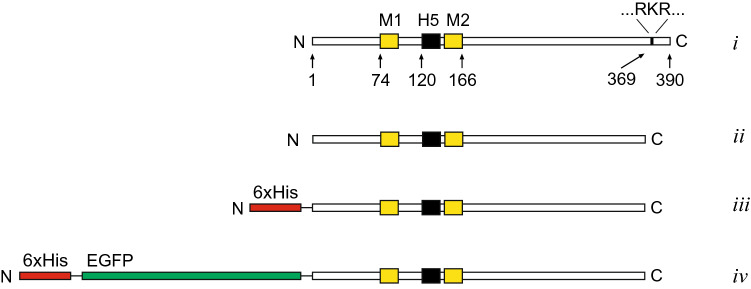
Schematic diagram of Kir6.2 variants. Illustration shows the Kir6.2 constructs used with the N- and C-termini deleted and/or fused with 6xHis and EGFP. Full length wild-type Kir6.2 contains 390 amino acids with two transmembrane segments (M1 and M2) and a pore-forming H5 segment (*i*). C-terminal truncated Kir6.2, Kir6.2ΔC26 (*ii*), N-terminal 6xHis tag fused with Kir6.2ΔC26, 6xHis-Kir6.2ΔC26 (*iii*), and 6xHis and EGFP chimera N-terminal fused with Kir6.2ΔC26, 6xHis-EGFP-Kir6.2ΔC26 (*iv*).

The RKR sequence in Kir6.2 is positioned near the C-terminus (AA369-371, Fig. 1) whereas in SUR1 the RKR sequence is positioned near nuclear binding fold-1 (Walker A motif)^36,37^. Mutation of RKR to AAA in SUR1 increases surface expression of sulfonylurea-sensitive K^+^ currents whereas the RKR to AAA mutation in Kir6.2 increases the expression of sulfonylurea-insensitive K^+^ currents due to enhanced Kir6.2 cell-surface expression^37^. Additionally, truncation of Kir6.2, where the last 26 or 36 amino acids (Kir6.2ΔC26 or Kir6.2ΔC36) containing the RKR signal were removed, facilitates expression of functional K^+^ channel activity that retains Kir6.2 ligand sensitivity in the absence of SUR1^26^. Therefore, the truncated mutants have been used to investigate ligand binding sites and other K_ATP_ channel structure–function relationships^26,27,38,39^.

Proper assembly of the K_ATP_ channel complex within the ER and subsequent regulated trafficking ensure normal orientation within the cell membrane^37^. However, little is known regarding the orientation of channels formed from truncated Kir6.2 mutants. This is particularly important since normal orientation is required to maintain ionic balance within the cell. Importantly, the K_ATP_ channel can conduct ions in both directions, yielding both inward and outward currents of different magnitudes^38,40–43^; however, the ligand sensitivity is specific to individual faces of the assembled channel. Many studies utilizing Kir6.2 mutants rely on whole-cell currents from mammalian cells or whole-cell or excised macropatch currents from *Xenopus* oocytes^26^ and thus may not be truly indicative of the population of normally oriented channels and may contain substantial ligand-insensitive background current resulting from abnormally oriented channels. In this work, we have investigated the effects of truncation of RKR on the plasma membrane orientation of Kir6.2 mutants expressed in *Xenopus* oocytes and mammalian cells using a combination of electrophysiological and immunocytochemical techniques.

## Materials and methods

### HEK293 culture

Human embryonic kidney (HEK293) cells, supplied by The American Type Culture Collection (ATCC), were cultured in minimum essential medium (MEM) supplemented with 10% fetal bovine serum (FBS) and 1% streptomycin and penicillin and incubated at 5% CO_2_, 37 °C. Media were changed every 2–3 days. Cells were split at 80–90% confluency using Puck's EDTA (140 mM NaCl, 5.5 mM KCl, 5.5 mM glucose, 4.2 mM NaHCO_3_, 0.5 mM EDTA, pH 7.40), followed by trypsin–EDTA with gentle rocking, and harvested with MEM. Cells were then centrifuged at 200 g for 2 min, resuspended into fresh MEM and placed into new flask at a 1:10 dilution. All media and additives were obtained from Invitrogen.

### *Xenopus leavis* oocytes

For collection of oocytes, large female *Xenopus laevis* were anaesthetized with 3-aminobenzoic acid methyl ester (1.5 g/L of water, Sigma) and handled using a previously established protocol^44^. Briefly, oocytes were removed from one ovary by laparotomy, the incision was sutured, and the animal was allowed to recover. Oocytes, stage V–VI, were defolliculated using collagenase A and injected using an Eppendorf transjector (Eppendorf, Hamburg, Germany) with 0.5–5 ng of mRNA/50 nL of sterile RNase-free water, encoding Kir6.2ΔC26 or Kir6.2ΔC26 together with SUR1. Oocytes were maintained in culture at a temperature of 19 °C, and experiments were performed 2–5 days after mRNA injection. Control oocytes were injected with 50 nL of sterile water.

### Preparation of expression plasmids for Kir6.2 mutants

For HEK293 cells, Kir6.2 mutants were originated from full-length human Kir6.2 cDNA template (courtesy of Dr. Joseph Bryan). The Kir6.2ΔC26 gene fragment was cloned into pcDNA4/HisMax-TOPO-TA vector using the TA cloning technique to construct a plasmid for expressing 6xHis-Kir6.2ΔC26 protein in mammalian cells. The sequences of the forward and reverse PCR primers were 5′-GGA TCC ATG CTG TCC CGC AAG GGC ATC-3′ and 5′-GGA TCC TCA GGC TGA GGC GAG GGT CAG-3′, respectively. The plasmid for expressing 6xHis-EGFP-Kir6.2ΔC26 in HEK293 cells was prepared by inserting the Kir6.2ΔC26 gene fragment from Kir6.2 cDNA into pET28a(+)/EGFP plasmid^45^ using forward (5′-ATA-GTC GAC AAA TGC TGT CCC GCA AGG GCA T-3′) and reverse primer (5′-ATG CGG CCG CAT CAG GCT GAG GCG AGG GTC AGA G-3′). After the pET28a(+)/EGFP-Kir6.2ΔC26 template was prepared, the EGFP- Kir6.2ΔC26 sequence was amplified using forward (5′-GGA TCC ATG GTG AGC AAG GGC GAG GAG-3′) and reverse (5′-GGA TCC TCA GGC TGA GGC GAG GGT CAG-3′), which was subsequently ligated into pcDNA4/HisMax-TOPO-TA vector to form the recombinant plasmid for expressing 6xHis-EGFP-Kir6.2ΔC26 in HEK293 cells. Plasmids were propagated in *E. coli* strain DH5α cultured in LB broth or on agar plates with 50 μg/mL ampicillin/kanamycin. Plasmids were purified using plasmid DNA isolation kits (Promega) and stored in Tris–EDTA (TE) buffer or Nanopure water at −20 °C. DNA quantity and purity was assessed using UV absorbance spectrophotometry (A_260_ and A_280_), agarose gel electrophoresis, DNA sequencing (University of Arizona Genetics Core Facility) and restriction digestion.

For oocytes, the cDNAs of mouse Kir6.2 (GenBank accession number D50581) and hamster SUR1 (GenBank accession number L40623) were subcloned into pBluescript II SK (Stratagene, La Jolla, CA), creating pB.mKir6.2 and pB.SUR1, respectively. Plasmid pB.mKir6.2Δ365–390 was generated by introducing a stop codon (R365Stop) into pB.mKir6.2 by site-directed mutagenesis using the QuikChange Mutagenesis kit (Stratagene). Capped mRNA was synthesized by in vitro transcription from linearized plasmids employing the mMESSAGE mMACHINE kit (Ambion, Austin, TX). The purified mRNA was dissolved in 10 mM Tris–HCl (pH 7.40) and stored in aliquots at −80 °C until use.

### Transfection of HEK293 cells and selection

Both transient and long-term expressions were initiated similarly. Cells were detached using trypsin and resuspended in fresh media at the following approximate densities: 1.8 × 10^4^, 1.0 × 10^5^, 4.2 × 10^5^, 2.8 × 10^6^, 1.3 × 10^6^, and 3.8 × 10^6^ per well for a 96 well plate, 24 well plate, 35 mm petri dish, 100 mm petri dish, 25 cm^2^ flask, or 75 cm^2^ flask, respectively. Cell densities were further optimized dependent upon cell type and plasmids. Cells were incubated for 24 h or until 80–90% confluency was reached for transient expression and 50–60% for long-term expression. At this point, transfection was performed using Lipofectamine 2000 (Invitrogen). Lipofectamine:DNA ratios were optimized by monitoring protein expression 48 h following transfection under varying ratios where an optimized ratio of 2.5 μL Lipofectamine: 1 μg of DNA was identified. Lipofectamine:DNA was added to cells in serum free Opti-MEM media, incubated for 4 h, followed by replacement with serum-supplemented media. Cells were incubated for 24–48 h to facilitate optimum transient protein expression. Long-term expressions were performed using Zeocin selection. Transfected cells were split after 48 h into new flasks or dishes, allowed to adhere to the surface for 12–24 h, and media replaced with Zeocin-doped media. Initial rounds of selection were typically performed in 24 well plates with a series of concentrations ranging from 50 to 500 μg/mL Zeocin. Media were changed every 2–3 days and cells were split as necessary. Typically 2–3 splits were performed prior to stable expression.

### RT-PCR

Expression of constructs was further verified via reverse transcriptase-PCR (RT-PCR). RNA was isolated from wild-type and transfected (6xHis-Kir6.2, 6xHis-EGFP-Kir6.2ΔC26, and 6xHis-Kir6.2ΔC26) cells using SV Total RNA isolation kit (Promega). RT-PCR was performed on isolated RNA using AccessQuick RT-PCR kit (Promega). Forward and reverse primers for 6xHis-EGFP-Kir6.2ΔC26 and 6xHis-Kir6.2ΔC26 were designed: 5′-GCG GCC GCA TGG GGG GTT CTC ATC ATC A-3′ (6xHis-Kir6.2ΔC26 Forward), 5′-TCT AGA TCA GGC TGA GGC GAG GGT-3′ (6xHis-EGFP-Kir6.2ΔC26), 5′-GCG GCC GCA TGG GGG GTT CTC ATC ATA-3′ (6xHis-Kir6.2ΔC26 Forward), and 5′-TCT AGA TCA GGC TGA GGC GAG GGT-3′ (6xHis-Kir6.2ΔC26 Reverse). Non-transfected controls utilized each set of primers. All primers were received from Integrated DNA Technologies and diluted into sterile water to prepare 100 μM stock concentrations. Working solutions of 10 μM were prepared for use in PCR.

### Immunocytochemistry

PentaHis-biotin conjugate (Mouse IgG1) (biotinylated anti-6xHis) was purchased from Qiagen (Catalog #34440). Streptavidin-fluorescein (Catalog #S869) and anti-GFP AlexaFluor 594 conjugates (Rabbit IgG) (Catalog #A21312) were purchased from Invitrogen. All antibodies were used as received. Cells were split onto coverslips 24–48 h prior to immunocytochemical staining. Immediately prior to staining, cells were washed twice with PBS, then fixed at room temperature using 4% (v/v) formaldehyde in PBS for 60 min. Cell fixation and all subsequent treatments were followed by 2–5 rinses with PBS. For permeabilized cells, 0.25% (v/v) Triton X-100 in PBS was added to the cells for 5 min following fixation. Non-specific adsorption was blocked by treatment with 5% FBS in PBS for 60 min. Expression and orientation of 6xHis-EGFP-Kir6.2ΔC26 were observed via staining with anti-GFP AlexaFluor 594 (2 µg/mL) in PBS for 60 min. Detection of 6xHis-Kir6.2ΔC26 required primary and secondary stains as follows: fixed cells were incubated with PentaHis-biotin conjugate in PBS for 60 min (0.2 µg/mL), followed by 1 µg/mL fluorescein-conjugated streptavidin for 60 min. Images were acquired using a using Nikon Eclipse TE300 inverted epifluorescence microscope with a 540/25 excitation filter and 620/60 emission filter for AlexaFluor 594 and 480/30 excitation filter and 535/40 emission filter for EGFP and fluorescein. Images were collected using a Cascade 650 front illuminated CCD camera or MicroMAX 512BFT back illuminated CCD camera (Roper Scientific, Tucson, AZ). MetaVue software version 1.0 (Universal Imaging, Downingtown, PA; https://www.moleculardevices.com/) was used to capture images and Image J^46^ was used to analyze all images.

### Electrophysiological recordings

Electrophysiological recordings were used to evaluate channel function and orientation. For HEK293 cells, recordings were collected using a HEKA EPC-8 using Pulse software version 7.0 (HEKA Elektronik Dr. Schulze GmbH, Germany; https://www.heka.com/about/about_main.html#smart-ephys). Pipettes were pulled from borosilicate glass and yielded a measured resistance between 4–6 MΩ. For whole-cell recordings, the extracellular solution (bath solution) was composed of (in mM): 138 NaCl, 5.6 KCl, 1.2 MgCl_2_·6H_2_O, 2.6 CaCl_2_, and 5 HEPES (pH 7.40). The intracellular solution (pipet solution) was composed of (in mM): 125 KCl, 1 MgCl_2_·6H_2_O, 30 KOH, 10 EGTA, and 5 HEPES (pH 7.15).

For oocytes, inside-out recordings of channel activity were obtained at a holding-potential of −80 mV and pipette solution containing (in mM): 140 KCl, 1.2 MgCl_2_·6H_2_O, 2.6 CaCl_2_, 5 HEPES at pH 7.40, and an internal (bath) solution consisting of (in mM) 140 KCl, 1 MgCl_2_·6H_2_O, 10 EGTA, 5 HEPES at pH 7.15. For outside-out recordings, the solutions were reversed. Recordings were made using an Axopatch 200 (Axon Instrument, CA). Channel records are displayed according to the convention that upward deflections denote outward currents and vice versa. The experiments were carried out at room temperature of 20–22 °C. All solutions were prepared in deionized water and filtered through a 0.2 µm pore-size filter prior to use. ATP was added as Mg^2+^-salt, and all reagents were of analytical grade and obtained from Sigma-Aldrich.

### Statistical analysis

For electrophysiological recordings, each group vs. control were compared using the Student’s t-test. All data are reported as mean ± SD.

## Results and discussion

The regulation of the K_ATP_ channel is complex. In addition to known small molecule modulators like ATP, ADP, sulfonylureas, etc., K_ATP_ channel function is regulated by expression, trafficking, and turnover in the plasma membrane. A key-regulatory element within the channel protein is the ER retention signal, RKR, in the C-terminal domain of both Kir6.2 and SUR1^37^. RKR serves as a point of control to ensure that only appropriately assembled channels with the correct stoichiometry and subunit composition traffic to the plasma membrane^37^.

Though truncated Kir6.2 mutants are known to form functional, ligand gated K^+^ channels, the effects of truncation on the orientation of Kir6.2 channels in the plasma membrane in the absence of SURx have not been explored. To further investigate the role of the RKR sequence in the regulation of K_ATP_ channels, the orientation of Kir6.2 mutants and the resulting functional implications, we utilized a combination of immunocytochemistry and electrophysiology of mammalian cells and *Xenopus* oocytes transfected with Kir6.2 mutants. For these studies, three constructs were prepared using wild-type Kir6.2 as template. Figure 1 shows a schematic representation of the (*i*) wild type Kir6.2; (*ii*) a C-terminal truncated Kir6.2, (*iii*) Kir6.2ΔC26 with an N-terminal hexahistidine (6xHis) tag (6xHis-Kir6.2ΔC26), and (*iv*) N-terminal EGFP-chimera of Kir6.2ΔC26 (6xHis-EGFP-Kir6.2ΔC26). These constructs were previously shown to generate an ATP-sensitive K^+^ current in oocytes and HEK cells^26,44,47^. Furthermore, the 6xHis tag allows for utilization of commercial antibodies raised against the tag to detect Kir6.2 mutants as well as facilitating potential purification of the protein in future applications^47^. Constructs were expressed in oocytes or HEK293, cells that lack native K_ATP_ channel background expression.

**Figure 2:**
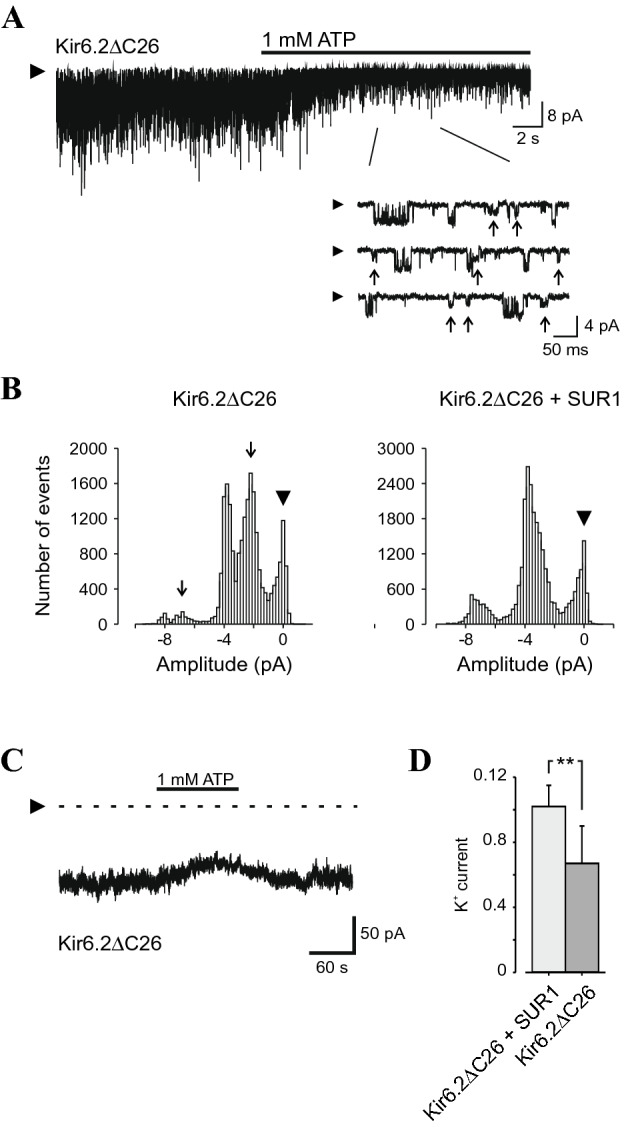
SUR1 affects orientation of Kir6.2 in the membrane. (**A**) Representative inside-out recording of channel currents from an oocyte injected with mRNA encoding Kir6.2ΔC26. Inset shows channel activity at an expanded time scale. Two populations of channel openings are observed, −3.8 ± 0.5 pA and −2.2 ± 0.3 pA (arrow). (**B**) Amplitude histogram of inside-out current traces from patches excised from oocytes injected with Kir6.2ΔC26 alone (*left*) or together with SUR1 (*right*). Data were obtained in the presence of 1 mM ATP in the bath solution (i.e. intracellular side). Arrow indicates additional population of channel openings seen in the absence of SUR1. A total of 22 × 10^3^ and 38 × 10^3^ events were obtained, respectively. (**C**) Representative recordings from Kir6.2ΔC26 channel activity from an outside-out patch, subsequently exposed to 1 mM ATP. (**D**) Summary of outside-out patches from oocytes injected with Kir6.2ΔC26 + SUR1 (*n* = 3) and Kir6.2ΔC26 (*n* = 4), and the effect of extracellularly 1 mM ATP expressed as the ratio between the current measured before and during ATP. Arrowhead indicated zero current level, error bars are ± SD and ** represents *P* < 0.01.

In *Xenopus* oocytes, addition of high concentrations of ATP revealed a population of K^+^ channels with a single channel amplitude of −2.3 ± 0.5 pA (Fig. 2A, *n* = 3), in addition to the population with an expected amplitude of −3.8 ± 0.3 pA (*n* = 3). Due to the inward rectification properties of Kir6.2, it is predicted that inverted channels with outward oriented N- and C-termini have a single channel amplitude, corresponding to an inverted rectification, of around −2 pA^26^, in good agreement with the observed second population of channel openings (Fig. 2B). In addition, when exposing outside-out patches from oocytes expressing Kir6.2ΔC26 to high concentration of extracellular ATP, we observed an inhibitory effect on channel activity (Fig. 2C), that likely results from exposure of the intracellular face to the extracellular milieu. It was impossible to determine with certainty if all channels, both normally oriented and abnormally inserted, were closed at that zero current level. Hence, there is a risk for underestimating the effect of extracellular ATP in these trials. However, when Kir6.2DC26 was co-expressed with SUR1, no effect of extracellular ATP is seen (Fig. 2D).

To evaluate the expression and orientation of Kir6.2 subunits, immunocytochemistry was performed on transfected HEK293 cells and non-transfected controls. In wild-type Kir6.2, both the N- and C-termini of the protein are found on the cytoplasmic face of the cell membrane^2^. Thus, no expression of N- or C- terminal epitopes should be observed when antibodies towards these regions are introduced to the exterior of the cell. Extracellular localization of the N-terminal 6xHis tag or EGFP was investigated in non-permeabilized cells, whereas total Kir6.2 membrane expression was evaluated in permeabilized cells, which allows access to antigens present on both the extracellular and cytoplasmic side of the membrane. Previous immunohistochemistry experiments have relied on hemagglutinin (HA) tags inserted into the Kir6.2 protein in the extracellular loop of the protein, facilitating detection of surface expression^37^. Importantly, these protocols were not extended to explore the possibility of inverted channels, thus potentially inverted channels were not studied. The protocol employed herein facilitates assessment of both protein expression and orientation of the varying constructs as outlined below.

**Figure 3:**
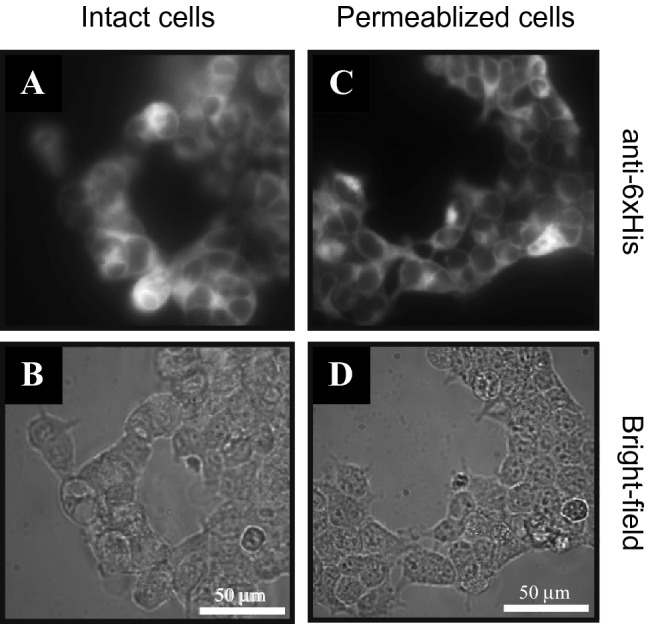
Immunocytochemistry of HEK293 cells transfected with 6xHis-Kir6.2ΔC26. Intact (**A**, **B**) and permeabilized (**C**, **D**) cells were utilized to explore the orientation of 6xHis-Kir6.2ΔC26 within the cell membrane. Biotinylated anti-6xHis was observed using streptavidin-fluorescein conjugate. Bright-field images (**B** and **D**) are provided for reference, and all images are on the same intensity scale.

Figure 3 shows typical fluorescence images obtained using HEK293 cells transfected with 6xHis-Kir6.2ΔC26. No intrinsic fluorescence at the wavelengths utilized was observed from the 6xHis-Kir6.2ΔC26 chimera, thus staining is required for visualization. For this task, biotinylated anti-6xHis was labeled with streptavidin-fluorescein conjugate. Expression of 6xHis-Kir6.2ΔC26 was observed in both intact and permeabilized cells, suggesting that accessible 6xHis moieties are present on both sides of the plasma membranes. In contrast, examination of wild-type, non-transfected HEK293 cells revealed no background membrane staining and thus low non-specific adsorption of reagents, demonstrating the reliability of cytochemical detection of inverted channels (Supporting Information, Fig. S1). For all immunocytochemistry, at least 10 successful transfections were analyzed, with at least 10 images per successful transfection.

**Figure 4:**
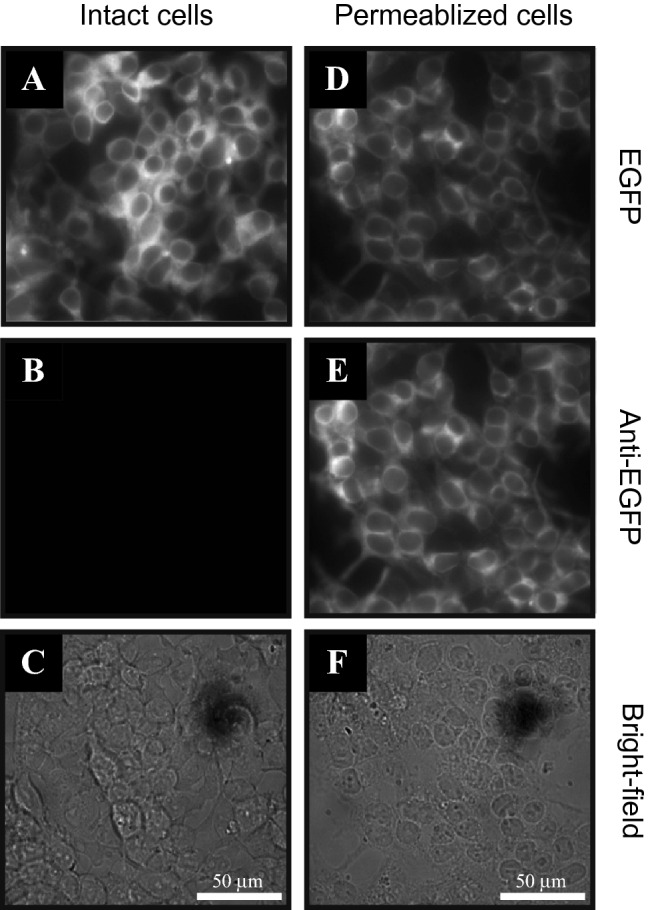
Immunocytochemistry of HEK293 cells transfected with 6xHis-EGFP-Kir6.2ΔC26. Intact (**A**–**C**) and permeabilized (**D**–**F**) cells were utilized to explore the orientation of 6xHis-EGFP-Kir6.2ΔC26 within the cell membrane. EGFP fluorescence (**A** and **D**) was observed under all conditions, whereas fluorescence resulting from anti-EGFP-AlexaFluor 594 conjugate was only observed in permeabilized cells (**B** and **E**). Bright-field images (**C** and **F**) are provided for reference. All fluorescent images are on the same intensity scale.

In contrast, expression of 6xHis-EGFP-Kir6.2ΔC26 exhibited a markedly different orientation profile. Figure 4 shows fluorescence images obtained using HEK293 cells transfected with 6xHis-EGFP-Kir6.2ΔC26. As anticipated, EGFP fluorescence was observed in all cells regardless of permeabilization. The orientation of EGFP was investigated using anti-EGFP-AlexaFluor 594 conjugate since spectral overlap of EGFP and fluorescein limited the use of streptavidin-fluorescein conjugates and streptavidin with red-shifted labels exhibited high non-specific staining. Upon staining, clear differences in the distribution of EGFP orientation were observed. No significant staining of EGFP was observed in intact cells, whereas AlexaFluor 594 signal was observed in all permeabilized cells. Combined, these data suggest that the EGFP moiety, and thus the N-terminus of the Kir6.2 chimera, is expressed on the cytoplasmic side of the cell membrane as is found in wild-type K_ATP_ channels, with little or no inversion of the channel proteins.

**Figure 5:**
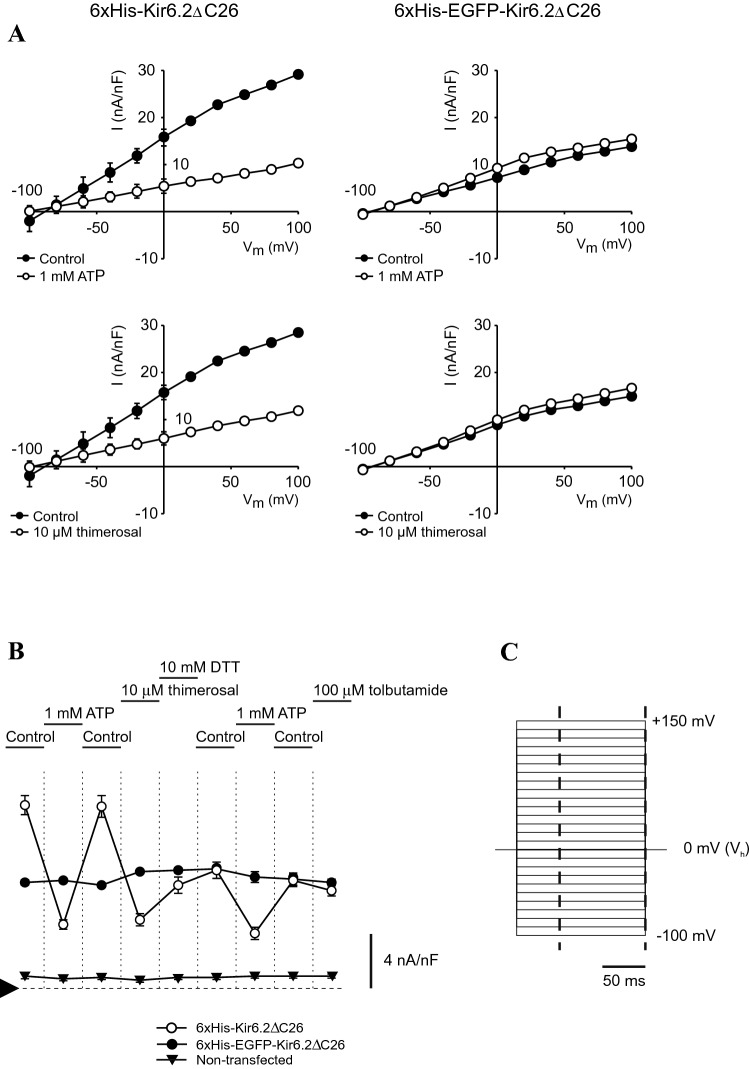
Whole-cell currents recorded from HEK293-cells expressing of different Kir6.2 variants. (**A**) Current–voltage (I–V) relationships of whole-cell currents in the presence of extracellular solution (denoted control), and 1 mM ATP or 10 µM thimerosal, as indicated. The cells were voltage-clamped at 0 mV (holding potential V_h_) for 25 ms prior to and following each pulse, and subsequently pulsed in steps of +10 mV for 150 ms, starting from −100 mV to +150 mV. (**B**), summary of normalized whole-cell currents of 6xHis-Kir6.2ΔC26 (open circles), 6xHis-EGFP-Kir6.2ΔC26 (filled circles), and non-transfected HEK293 (filled triangles) recorded at 0 mV. Whole-cell currents were normalized using membrane capacitance to compensate for variations in cell size. Each recording represents consecutive exposures to control, 1 mM ATP, control, 10 µM thimerosal, 1 mM DTT, control, 1 mM ATP, control, and finally 100 µM tolbutamide. Arrowhead indicates zero current level, and error bars are ± SD. *n* = 10 for each cell type. (**C**) Mean currents were measured from 50 to 150 ms (dashed lines) and were plotted *versus* applied potential. Background current measured in transfected HEK293 cells was subtracted from mean currents, and plotted under the assumption that *E*_*K*_ is −83 mV in the solutions used. Only −100 mV to +100 mV is presented, in +20 mV incremental steps, in the I–V relationships in (**A**).

To correlate the function of Kir6.2ΔC26 subunits outlined above with membrane orientation, electrophysiological characterization of HEK293 cells transfected with either 6xHis-Kir6.2ΔC26 or 6xHis-EGFP-Kir6.2ΔC26 was performed using whole-cell recordings (Fig. 5). Though whole-cell recordings monitor the net macroscopic current for the entire cell, the capability to monitor K^+^ flux in the presence and absence of membrane side-specific Kir6.2 modulators facilitates investigation of orientation. Non-transfected and transfected HEK293 cells were chosen for these experiments as they do not natively express K_ATP_ channels and typically have low total ion currents prior to transfection. Whole-cell currents were measured when the cells were immersed in extracellular solution, followed by exposure to 1 mM ATP on the cytoplasmic side, a concentration sufficient to inhibit >90% of K^+^ flux via Kir6.2ΔC26^3,4,26,47^. As seen in Fig. 5A, B, exposure to 1 mM ATP reduced the net current by ca. 60% in cells transfected with 6xHis-Kir6.2ΔC26. Importantly, the binding site for ATP resides on the cytoplasmic side of the wild-type K_ATP_ channel, and ATP does not readily cross the cell membrane. When ATP was removed from the solution, whole-cell currents returned to their previous values. Thus, reversible inhibition by ATP is supportive of inverted channels within the membrane. In a similar experiment performed on cells transfected with 6xHis-EGFP-Kir6.2ΔC26, no statistically significant decrease in K^+^ currents were observed. However, larger whole-cell currents were observed for cells expressing 6xHis-Kir6.2ΔC26. Thus, the current differences observed are likely due to increased protein expression and/or trafficking of 6xHis-Kir6.2ΔC26 compared to the larger 6xHis-EGFP-Kir6.2ΔC26. Interestingly channel activity was retained even in the presence of physiological Ca^2+^ levels in the extracellular solution. Wild-type K_ATP_ channels are phosphorylated by intracellular PKA and PKC, exposed to adequate cytoplasmic milieu such as low Ca^2+^, and associated with phosphatidylinositols, which may also lower currents.

To further explore this phenomenon, cells were exposed to thimerosal, a membrane impermeant oxidizing agent. Previous studies showed that exposure of the intracellular face of wild-type K_ATP_ channels residing in excised membrane patches to thimerosal resulted in loss of channel activity that could be partially reversed upon exposure to a suitable reducing agent, e.g. DTT^48^. Exposure of cells transfected with 6xHis-Kir6.2ΔC26 to thimerosal resulted in a ca. 60% reduction in current, approximately 50% of which was restored upon exposure to DTT, in good agreement with previous work^48^, at which point whole-cell currents were unchanged upon exposure to buffer (Fig. 5). Conversely, no loss of channel activity was observed in cells transfected with 6xHis-EGFP-Kir6.2ΔC26 upon exposure to thimerosal nor was activity enhanced upon exposure to DTT. When these same cells were exposed again to ATP, a ca. 60% decrease in activity was observed for cells expressing 6xHis-Kir6.2ΔC26. Exposure to inhibiting concentrations of tolbutamide yielded no statistically significant differences in whole-cell currents for either construct, an expected result since SUR1 is not expressed in these cells. For non-transfected, wild-type HEK293 control cells, sustained low level currents, likely from endogenous ion channel expression, were observed in all solutions tested with no statistically significant changes upon application of K_ATP_ channel modulators (Fig. 5B), supporting the low K_ATP_ background conductance in these cells. HEK293 cells endogenously express P2Y receptors^49^, which could be potentially activated to decrease K_ATP_ channel activity by reducing PIP_2_ near the K_ATP_ channel^39,50^. However, since no effect of extracellular applied ATP on K^+^ current was seen in cells expressing 6xHis-EGFP-Kir6.2ΔC26, the potential effect of P2Y activation is likely small. Finally, the current measured for 6xHis-Kir6.2ΔC26 under these conditions is approximately 2 × that for 6xHis-EGFP-Kir6.2ΔC26. Since the mean channel conductance is similar for both constructs, the observed difference in current likely results from higher expression and/or membrane trafficking of the significantly smaller 6xHis-Kir6.2ΔC26.

Though immunocytochemical staining alone might reveal non-functional monomers or oligomers of Kir6.2 that are inadvertently transported to the plasma membrane, the ligand-modulated K^+^ currents that are observed upon extracellular addition of ligands strongly support the functional expression of an inverted K^+^ channel within the cell membrane. Combined, the electrophysiological and immunocytochemical data support the hypothesis that Kir6.2ΔC26 transfected into mammalian cells results in a sub-population of inverted ion channels in the plasma membrane that retain ligand sensitivity and ion conductance.

**Figure 6:**
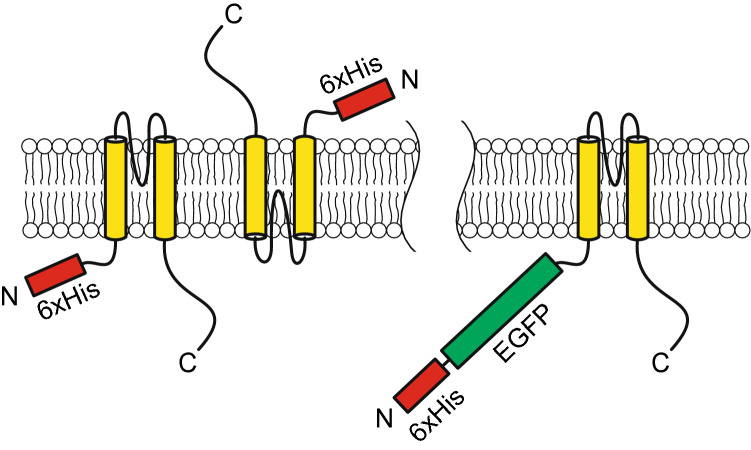
Schematic representation of protein orientation. Immunocytochemical and electrophysiological data support a model where Kir6.2ΔC26, which lacks native ER retention and membrane trafficking signals, is abnormally inserted in the plasma membrane (*left*), suggesting abnormal trafficking. Conversely, inclusion of a large, water-soluble domain on the N-terminus imparts a sufficient energy barrier such that all the proteins are inserted in a unidirectional manner, overcoming the lack of ER retention and trafficking signals (*right*).

In wild-type K_ATP_ channels, the RKR retention signal in both Kir6.2 and SUR1 at the cytoplasmic face provides a quality control check that ensures the channels are properly assembled in the ER before further trafficking to the Golgi apparatus^36,37^. Truncated Kir6.2 mutants that lack the RKR sequence are capable of trafficking to the membrane allowing this quality control checkpoint to be bypassed. Our data support the hypothesis that not only are Kir6.2 homologous channels able to exit the ER and traffic through the Golgi apparatus to the plasma membrane, but that they may form functional channels that are inserted into the plasma membrane in an inverted orientation. Interestingly, the attachment of a large, water-soluble protein domain, EGFP in this case, located at the N-terminus significantly modulates the orientation of the protein, resulting in normal orientation and regulation of the channel within the plasma membrane. We hypothesize that these differences in orientation result from changes in post-translation processing of the proteins within the ER. EGFP serves to significantly change the orientation of the protein, most likely by introducing a large energetic barrier for protein orientation, resulting in unidirectional insertion (Fig. 6).

Lastly, there are many potential binding sites for PIP_2_ on Kir6.2, several of which are located in the C-terminus^39^. The mechanism for how PIP_2_ interacts and affects channel activity is not entirely clear, but a possible effect with the C-terminal truncation of Kir6.2 is that the channel is activated partly because of its altered PIP_2_ interaction. In that case, it could also explain why truncated Kir6.2 can be open even if abnormally inserted into the plasma membrane. A reasonable control would have been full-length Kir6.2. However, this construction does not generate K^+^ conductance without co-expression of SUR1, and hence, co-expression with SUR1 results in truncated Kir6.2 being normally inserted. Shorter truncations of Kir6.2, like C14 and C18, resulted in low K^+^ conductance^26^, so it is likely that the RKR (AA369-371) included in the deletion C26, and no other positively charged amino acids in the c-terminal region (Arg-377, Arg-379, and Arg-381) are the main explanation for abnormal insertion. The impact of the His-tag fused to Kir6.2ΔC26 (construct *iii*, Fig. 1) is difficult to assess, but it appears not to be sufficient to orient the Kir in the normal position as the inclusion of EGFP does (construct *iv*, Fig. 1).

The potential biological significance of these observations is currently unknown. However, mutations in K_ATP_ channels that alter trafficking to the plasma membrane have been linked to PHHI^32^. It is possible that as yet unknown trafficking, assembly and orientation mechanisms may play a role in K_ATP_ channel defects associated with abnormal physiological function. The fact that a protein can be translated, trafficked and expressed in a cell membrane within a mammalian cell line in an inverted fashion presents a number of intriguing possibilities for altered biological function that should be further investigated.

## Conclusion

The data presented in this research report is the first observations of inverted ATP-sensitive K^+^ channels within cellular expression models. Truncated mutants of Kir6.2, when expressed alone, were found to yield randomly inserted channel complexes, both normal and abnormal insertions, that were modulated in a cell surface specific manner upon application of ligands to the extracellular side of the channel. Abnormal channel population was not seen when co-expressed with SUR1. Fusion expression of a large, water-soluble protein domain like EGFP, to the cytoplasmic N-terminal domain of the channel resulted in channels that were correctly oriented within the cell membrane. We postulate that the lack of the RKR ER retention signal in the truncated Kir6.2 mutants used in our experiments prevents proper quality control and that the RKR sequence plays a key role in channel orientation in addition to channel assembly during the translation and trafficking processes. While the biological significance remains unknown, the existence of abnormally inserted channels presents a number of interesting possibilities for defective biological function and warrants further investigation. Further, these data suggest the need for caution in interpreting results from cellular studies relying on truncated Kir6.2 mutants and other truncated sequences, as well as potential effects on protein orientation of membrane proteins tagged with fluorescent proteins. Finally, expression of inverted channels may present unique opportunities to investigate compounds that may modulate Kir6.2 but are membrane impermeant, thus avoiding the need for injection or other delivery platforms to study the effects of such compounds.

## Supplementary Information

Supplementary Information.
