# Supplementary figures and images for: Expression of truncated Kir6.2 promotes insertion of functionally inverted ATP-sensitive K+ channels
Source: Sci Rep. 2021 Nov 2;11:21539. doi: 10.1038/s41598-021-00988-y (PMC8564548; doi:10.1038/s41598-021-00988-y)

Figure SI-1

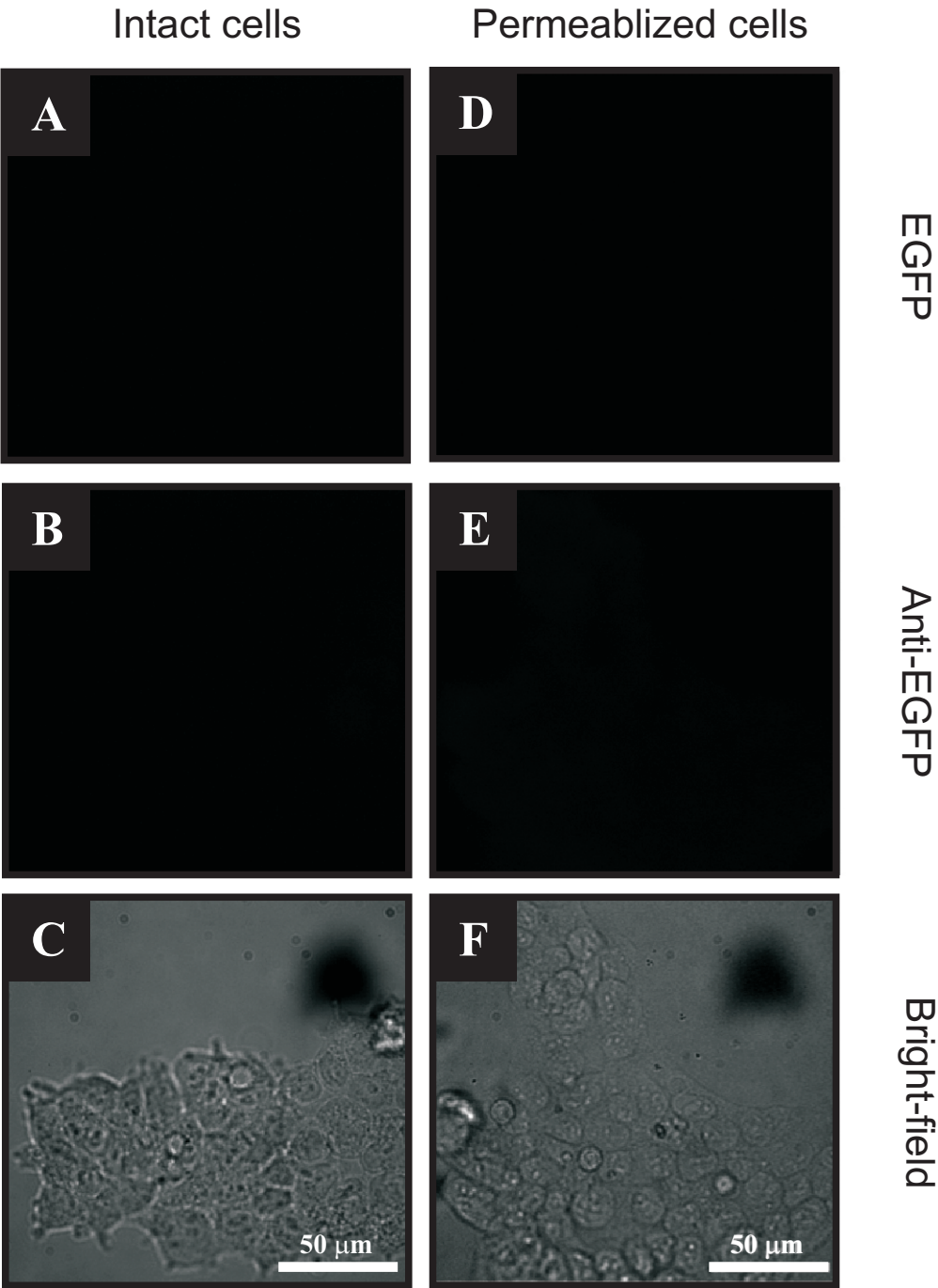

Supplement: Supplementary file 1 — Supplementary Information. [file 41598_2021_988_MOESM1_ESM.pdf]
